# *PPP3R1* Promoter Polymorphism (Allelic Variation) Affects Tacrolimus Treatment Efficacy by Modulating E2F6 Binding Affinity

**DOI:** 10.3390/biomedicines12122896

**Published:** 2024-12-19

**Authors:** Xinyi Zheng, Shengying Qin, Mingkang Zhong, Qinxia Xu, Cong Huai, Xiaoyan Qiu

**Affiliations:** 1 Department of Pharmacy, Huashan Hospital, Fudan University, 12 Middle Urumqi Road, Shanghai 200040, China; 17111030005@fudan.edu.cn (X.Z.);; 2 Bio-X Institutes, Key Laboratory for the Genetics of Developmental and Neuropsychiatric Disorders (Ministry of Education), Shanghai Jiao Tong University, No. 1954 Huashan Rd, Shanghai 200030, China; 3 Department of Pharmacy, Zhongshan Hospital, Fudan University, Shanghai 200032, China

**Keywords:** tacrolimus, calcineurin, single nucleotide polymorphism, E2F6, PPP3R1

## Abstract

Background: Tacrolimus is widely used as a first-line immunosuppressant in transplant immunology; however, its clinical application is constrained by the narrow therapeutic index and considerable interindividual variability. In this study, we identified the potential regulatory role of a novel *PPP3R1* promoter polymorphism, rs4519508 C > T, in the tacrolimus pharmacodynamic pathway. Methods: Dual-luciferase reporter assays and bioinformatic analysis were applied to assess the impact of allelic variation. Electrophoretic mobility shift assays (EMSA) validated the altered binding of transcription factors. Quantitative real-time PCR (qRT-PCR), enzyme-linked immunosorbent assay (ELISA) and Western blots were used to determine the immunosuppressive effect of tacrolimus. Results: Assays revealed that rs4519508 C > T markedly enhanced *PPP3R1* promoter activity. EMSA assays validated the binding of E2F6 to rs4519508 C (wild-type) and the binding was significantly weaker to the rs4519508 T (mutant-type). The overexpression of E2F6 significantly reduced the transcriptional activity and expression of PPP3R1 when the rs4519508 site presented as major C allele, an effect that was not observed with the rs4519508 T allele. Furthermore, the downregulation of E2F6 raises the level of downstream immune cytokines inhibited by TAC. Conclusions: This study proposed that E2F6 suppresses the expression of PPP3R1, while rs4519508 C > T impairs the binding of E2F6, and thus elevates the level of PPP3R1, so that the inhibition of the downstream immune cytokines by TAC is attenuated. Our findings reported the potential regulatory role of a novel polymorphism, *PPP3R1* rs4519508 C > T, which may serve as pharmacodynamic-associated pharmacogenetic biomarker indicating individual response variability of tacrolimus, and thus aid the clinical management of transplant immunology.

## 1. Introduction

Tacrolimus (TAC, FK506), a calcineurin inhibitor (CNI), has been widely used as the first-line immunosuppressive agent for kidney transplantation and has greatly improved the short- and long-term survival of kidney grafts [1,2,3]. However, TAC is featured by its narrow therapeutic index, and the pharmacokinetic (PK) parameters and pharmacodynamic (PD) responses are heterogeneous dramatically among individuals [2,3,4,5,6]. Currently, therapeutic drug monitoring is routinely used for dosage adjustment in clinical practice to achieve a recommended therapeutic target range for TAC [7]. However, the inter-individual variability of TAC safety and efficacy is still of great concern. As reported, the PD variability of TAC increases the incidence of acute rejection, acute nephrotoxicity, infection, and abnormal renal allograft function after transplantation (B II) [1,6,8,9,10,11].

The immunosuppressive effect of TAC exerts mainly through inhibiting the calcineurin (CaN) signaling pathway [1]. TAC forms a complex with the specific immunophilin, FK506 binding protein 12 (FKBP12) [12] in T cells and acts on CaN, thus inhibiting the dephosphorylation and nuclear translocation of the nuclear factor of activated T cells (NFAT), and blocking the recruitment of immune cytokines involved in T-cell activation, such as interleukin-2 (IL-2) and granulocyte-monocyte colony stimulating factor (GM-CSF), subsequently [1,13,14,15]. CaN is comprised of two subunits, a catalytic (CNA) and a regulatory subunit (CNB) [8,16]. The CNA gene family encompasses three members (*PPP3CA*, *PPP3CB*, and *PPP3CC*), while the CNB gene family consists of two members (*PPP3R1* and *PPP3R2*) [15,16]. Of these five coding genes, *PPP3CA*, *PPP3CB*, and *PPP3R1* are extensively expressed in human T and B cells [3,17,18] and are believed to contribute to the pharmacological effects of TAC.

Several genetic polymorphisms in the FKBP-CaN-NFAT pathway have been reported to influence the clinical outcomes of TAC in post-transplant patients. Association studies found that *FKBP1A* rs6041749 was correlated with post-transplant allograft function [19], and *NFATC2* rs2426295 was significantly correlated with acute rejection after renal transplantation [20]. A few critical SNPs, *PPP3CA* rs45441997, rs3804358, *PPP3CB* rs3763679, *PPP3R1* rs1868402, and *IL2* rs2069763, were referred to as “highly recommended” candidate variants associated with TAC response or efficacy [5].

Our previous study in 140 Chinese Han renal transplant patients who receiving TAC treatment also identified several SNPs in gene *PPP3R1* that influence TAC response: rs875 (*p* = 0.037) and rs4347819 (*p* = 0.045) associated with allograft function [3], and rs7560138 (*p* = 0.024) correlated with the incidence of acute rejection [19]. In addition, we revealed that *PPP3R1* rs875 T > C influences TAC efficacy in idiopathic membranous nephropathy (IMN) patients through altering the binding affinity of miR-582-5p [15]. Clinical association studies have shown sufficient relevance of variations in PD pathway on TAC efficacy, while functional validation is needed to further elucidate the regulatory mechanism.

Linkage disequilibrium (LD) analysis combined with in silico prediction captured a non-coding promoter polymorphism rs4519508 C > T in gene *PPP3R1* that was in strong LD with the validated functional variant, rs875 (*r*^2^ > 0.9) [15], and located within several TF motifs. Previous studies have shown the importance of studies on LD SNPs with critical SNPs in the identification of causal mutations and genetic biomarkers [21,22,23,24,25,26]. To investigate the regulatory mechanism of potentially functional SNPs, we conducted in silico prediction and in vitro functional assays to evaluate the effects of the mutations on luciferase activity, *PPP3R1* mRNA expression, and the immunosuppressive effect of TAC. The aim of this study is to elucidate the functional role, if any, of polymorphisms in *PPP3R1* in the FKBP-CaN-NFAT signaling pathway and the immunosuppressive effect of TAC.

## 2. Material and Methods

### 2.1. Cell Culture

Human epithelial kidney 293 T cells (HEK 293 T) and Jurkat T cells (an immortalized human T lymphocyte cell line) were maintained in RPMI-1640 medium (Gibco, Carlsbad CA, USA) enriched with 10% fetal bovine serum (Gibco, Carlsbad, CA, USA). Cultures were incubated in a humidified environment with 5% CO_2_ at a temperature of 37 °C. Transfection of cells was conducted in 24-well plates.

### 2.2. Jurkat T Cell Activation

Jurkat T cells were centrifuged at 1000 rpm for 5 min and resuspended in 0.5–1 mL of fresh complete RPMI medium and planked with a cell density of 2 × 10^5^ each hole in round-bottom 96-well culture plates. After recovery and rewarming for 5 min at 37 °C, the cells were stimulated with antibodies against CD3 and CD28 (anti-CD3, 1 μg/mL; anti-CD28, 5 μg/mL). After stimulation, cells were placed on ice, washed three times with phosphate buffered saline (PBS), and resuspended in complete RPMI medium.

### 2.3. Plasmid Construction

The promoter region fragment (956 bp) of *PPP3R1* containing the rs4519508 major C allele was cloned into the *MluI* and *BglII* sites of the dual luciferase reporter gene vector pGL3-basic using T4 DNA ligase (New England BioLabs, Boston, MA) to obtain the pGL3-rs4519508C plasmid. The 3′UTR region fragment (401 bp) of *PPP3R1* containing rs875 T was cloned into the *XbaI* and *BamHI* sites of the pGL3-promoter vector to build the pGL3-rs875T plasmid. The Q5^®^ Site-Directed Mutagenesis kit (E0554; NEB, Ipswich, MA, USA) was used to construct site-directed mutant recombinants, named pGL3-rs4519508T and pGL3-rs875C, with the mutagenic primer designed by NEBaseChanger™. Next, the ClonExpress^®^ II One Step Cloning Kit (C112) (Vazyme Biotech Co., Ltd. Nanjing, China) was used to reassemble fragments containing rs4519508 C/T and rs875 T/C into one reporter vector at the *XbaI* site, respectively, and obtained the pGL3-rs4519508C-rs875T, pGL3-rs4519508T-rs875T, pGL3-rs4519508C-rs875C, and pGL3-rs4519508T-rs875C plasmids (6157 bp). The information of the primer pairs is provided in Supplementary Table S1. Supplementary Figures S1 and S2 provide the whole workflow of the plasmid construction, and additional details are illustrated in Appendix A.

Further, the overexpression vectors of four transcription factors, STAT1, E2F6, E2F4, and ETS1, were constructed by cloning the coding region amplified from human cDNA into the pcDNA3.1 vector and verified by Sanger sequencing. The primer pairs used are presented in Supplementary Table S2.

### 2.4. Transient Transfection and Dual-Luciferase Reporter Gene Assays

HEK 293 T cells were transiently co-transfected in 24-well plates with 1 μg of the recombinant reporter plasmids and 0.05 μg of the pRL-TK Renilla luciferase control vector (Promega, Madison, WI, USA), using 2 μL of Lipofectamine 2000 (Invitrogen, Carlsbad, CA, USA) and jetPEI^®^ (Polyplus, Illkirch, France), according to the manufacturers’ protocols. An additional 48 h of incubation was conducted after transfection, then we measured the level of firefly luciferase activity using the Dual-Luciferase Reporter Assay System (Promega, Madison, WI, USA). The relative luciferase activity was calculated by the ratio of Renilla luciferase activity to firefly luciferase activity. All assays were executed in triplicate to ensure reproducibility.

### 2.5. In Silico Bioinformatic Prediction

The sequence of rs4519508 with 10 nts both sides were applied to in binding factors prediction silico. Both C and T alleles were set as seeds. The R packages used included biomaRt [27,28], BSgenome [29], TFBSTools [30], JASPAR2018 [31], and Biostrings [3,32]. The minimum combination score for transcription factor identification was set as 80%. The factors captured were filtered through comparing with the ChIP-seq data in human downloaded from the Cistrome Datasets Browser [33,34] (http://cistrome.org, accessed on 1 February 2022) [3]. Differential binding transcription factors were screened out by comparing the difference between the predictions of the two sequences [3]. The screening criterion involved TF gain/loss, binding site alteration, or Δcombination score ≥ 0.05 [3]. In addition, STRING (https://string-db.org/, accessed on 13 March 2022) was used to depict the protein–protein interaction network for *PPP3R1*.

### 2.6. Quantitative Real-Time PCR Analysis (qRT-PCR)

Total RNA was extracted from treated cells by using TRIzol reagent (Invitrogen, Carlsbad, CA, USA), then reverse transcription was conducted using the PrimeScript^®^ kit (RR036A; TaKaRa, Kusatsu, Shiga Prefecture, Japan). Agarose gel electrophoresis was used to determine the integrity of each RNA sample.

Samples were analyzed using standard quantitative real-time PCR (qRT-PCR). The primers were provided in Supplementary Table S3. SYBR Premix Ex Taq Kit (RR820A; TaKaRa, Kusatsu, Shiga Prefecture, Japan) and a StepOne Plus RealTime PCR System (Applied Biosystems, Foster City, CA, USA) were implemented. The total volume of the amplification system was 20 µL per action, containing 10 μL of SYBR Green Master Mix, 0.4 µL of ROX Reference Dye, 0.4 μL of each primer (10 μmol), 1 μg of cDNA, and 7.8 μL of nuclease-free water.

The amplification conditions were pre-denaturation at 95 °C for 5 min, followed by 40 cycles of amplification (95 °C for 10 s and 60 °C for 30 s). Melting curve analysis was performed from 60 °C to 95 °C by reading the plate every 0.3 °C. Relative target mRNA levels were calculated using the 2^−ΔΔCT^ method. Experiments were performed at least three times. Agarose gel electrophoresis and DNA sequencing were conducted to validate the amplification products. The ratio between certain mRNAs and GAPDH was set as reference.

### 2.7. Electrophoretic Mobility Shift Assay (EMSA)

Nuclear extracts from heat-shocked (1 h at 42 °C) Jurkat T cells were prepared using a Nuclear/Cytoplasmic Protein Extraction Kit (Viagene Biotech, Changzhou, China, SINP001) [35]. Protein concentrations were quantified with a Enhanced BCA Protein Assay (Viagene Biotech, CHEM001, Changzhou, China). EMSA assay was performed via Non-Radioactive EMSA Kits with Biotin-Probes [36] (Cool-Shift, Viagene Biotech, Changzhou, China, SIDET001). In brief, for binding assay, 3 mg (low) or 6 mg (high) concentration of total nuclear extract were co-cultured with 100 fmol of biotinylated annealed oligonucleotides in 20 mL of total reaction volume. For supershift assays, 2 mg of the E2F6 antibody (ab53061, Abcam, Cambridge, UK) was incubated with the treated reaction mixture for an extra 30 min on ice. DNA–protein complexes were isolated in the environment of 6% native polyacrylamide gels incubated in 0.5 × TBE (Tris-Borate-EDTA)(Beyotime, Wuhan, China) buffer, transferred to nylon membranes (Thermo Fisher Scientific, Waltham, MA, USA), and detected by chemiluminescent detection methods after UV cross-linking. The differences between each group were calculated using one-way ANOVA with SNK a posteriori comparison of means.

### 2.8. Western Blotting

Protein of PPP3R1 was extracted using RIPA lysis buffer, added with 1%PMSF (100 mM), containing protease inhibitors (Beyotime, Wuhan, China). Protein concentrations were determined by the BCA protein assay (Thermo Fisher Scientific, Waltham, MA, USA). The proteins were isolated with sodium dodecyl sulfate-polyacrylamide gel electrophoresis and were transferred to polyvinylidene fluoride membranes (Millipore, MA, USA). The membranes were blocked by 5% nonfat milk and incubated overnight at 4 °C with a primary antibody against human PPP3R1 (MAB1348; R&D Systems, Minnneapolis, MN, USA), E2F6 (UPA00361; Gene universal, Newark, DE, USA), and Tubulin antibody (Bioworld, BS1482M, Bloomington, MN, USA). Afterward, membranes were incubated with HRP-conjugated secondary antibody (A0208; Beyotime, Wuhan, China) for 1 h at room temperature. Chemiluminescence reagent (Millipore, Bedford, MA, USA) was used to capture and assess signals. Relative PPP3R1 protein expression was quantified by densitometry using ImageJ software (version 1.46 r; NIH) [37].

### 2.9. Establishment of Stable Cell Lines

The lentivirus package was constructed by Integrated biotech solutions Inc. (Shanghai, China). For lentivirus production, 293T cells were co-transfected with pCDH-MSCV-MCS-EF1-GFP-Puro vectors, psPAX2, and pCMV-VSVG, by use of the PEI transfection method. After transfection, the virus supernatant was collected twice, at 48 and 72 h, respectively. After collection, the supernatant was filtered through a 0.45 μm filter, and centrifuge in a 40 mL ultracentrifuge tube at 4 °C and 72,000 g/min. Cells were cultivated at 30% confluency (5 × 10^4^ cells/well) in 24-well plates. After 12–20 h, cells were infected with the virus pCDH-MSCV-MCS-EF1-GFP-puro (E3885). The virus titer was 3.78 × 10^8^ TU/mL, and the MOI (multiplicity of infection) value was 1.32 μL. A volume of 2.5 μL of 1 mg/mL polybrene (Sigma: H9268, St. Louis, MO, USA) was added to each well, and the final concentration of polybrene in the cell sample was 5 μg/mL. We constructed the recombinant LVs, which expressed E2F6 shRNA (LV-E2F6-shRNA). LV-expressing shRNA negative control expressed scrambler siRNA as shRNA control (LV-shRNA-NC). Cells were seeded into 24-well plates at 3 × 10^4^ cells/well and cultured overnight. Then, cells were transduced with the corresponding lentiviral vector. After 24 h, the transduced cells were diluted at 1:100 and plated onto 100 mm culture dishes with 5 μg/mL polybrene (IBSBIO, Shanghai, China) and 2 μg/mL puromycin (IBSBIO, Shanghai, China) for 2 weeks. Clones displaying puromycin resistance and expressing EGFP were selected and expanded. Stable knockdown of E2F6 was confirmed by RT-qPCR and Western blot analysis. Transduction was performed according to the manufacturer’s protocols.

### 2.10. Detection of IL-2 and GM-CSF Production

After infection with LVs, Jurkat T cells were treated with (or without) TAC at a concentration of 0.008 ng/mL for 30 min, incubated with anti-CD3 (1 μg/mL) and anti-CD28 (5 μg/mL) antibodies for stimulation [15]. After 24 h of stimulation, supernatants were collected and analyzed for IL-2 and GM-CSF levels using quantitative real-time PCR (qRT-PCR) and enzyme-linked immunosorbent assay (ELISA) kits (MultiSciences, Hangzhou, China).

### 2.11. Statistical Analysis

The results are presented by mean ± SD. Student’s *t*-test and standard one-way ANOVA were applied for repeated measurements. GraphPad Prism 8.3.0 software was utilized for data processing (San Diego, CA, USA). Significance was defined as *p* < 0.05.

## 3. Results

### 3.1. Rs4519508 (C > T) and rs875 (T > C) in Strong LD Increased Luciferase Reporter Activity Comparably

**Figure 1:**
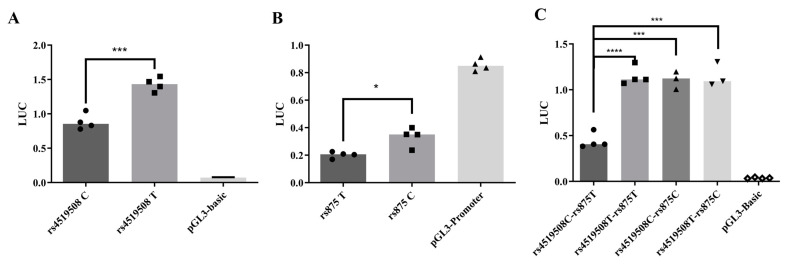
DLR assays comparing effects of LD SNPs (rs875 and rs4519508) in *PPP3R1*. (**A**,**B**) Effects of rs4519508 and rs875 on transcriptional activity of PPP3R1. (**C**) Effects of combined rs4519508C/T-rs875T/C on transcriptional activity of PPP3R1. Columns 1–4 present luciferase activity of 293T transfected by pGL3-basic containing fragments of both rs4519608C(T) and rs875T(C); column 5 indicates luciferase activity of 293T transfected by pGL3-basic. (*n* = 3) ****, *p* < 0.0001, ***, *p* < 0.001, *, *p* < 0.05. *p*-values were calculated using Student’s *t*-test.

We used the dual-luciferase reporter gene system (DLR) to investigate the influence of rs4519508 (C > T) on the transcription levels of *PPP3R1*. Luciferase activity was significantly upregulated for pGL3-rs4519508T compared to the pGL3-rs4519508C (*p* < 0.01, Figure 1A). The results demonstrated that the mutation from C to T at the rs4519508 site upregulated the transcription level of *PPP3R1*.

To compare the influence of the two LD SNPs, rs875 and rs4519508 (*r*^2^ > 0.9), within *PPP3R1* on transcriptional regulation, we used DLR system to conduct parallel experiments. The results showed that pGL3-rs875C had significantly higher luciferase activity than pGL3-rs875T (*p* = 0.011, Figure 1B). DLR experiments comparing the luciferase activity of four constructed recombinant plasmids, pGL3-rs4519508C, pGL3-rs4519508T, pGL3-rs875T, and pGL3-rs875C, showed an increase in transcriptional activity in both LD SNPs, and the increased activity induced by rs4519508 C > T was comparatively greater than that induced by the mutation rs875 T > C (Figure 1A,B).

To further investigate the synergistic or antagonistic effect between rs4519508 and rs875, we constructed four recombinant plasmids containing both LD SNPs. As shown in Figure 1C, the single mutation rs4519508 C > T or rs875 T > C combined with the wild-type at the other site significantly enhanced luciferase activity (*p* < 0.001). Supplementary Table S4 shows the *p*-values and significance between different groups. The effects of two single mutations on gene expression and transcriptional activity were similar, whereas the increase induced by a single mutation rs4519508 C > T was slightly higher than that of the single mutation rs875 T > C. In comparison to the single mutation situation, the two-site mutation group had a slightly higher expression level of luciferase activity.

### 3.2. In Silico Functional Prediction Found SNP Allelic Variation Change the Binding Affinity of TF

As mutations within binding sites in the core promoter region may alter the binding of transcriptional regulatory elements and lead to the transcriptional inhibition or activation of genes [38,39,40], here we used in silico TF prediction to predict TFs that bind the sites containing rs4519508 (C > T) in the *PPP3R1* promoter region. There are five TFs, E2F4, E2F6, ERG, ETS1, and STAT1, showing differential binding affinity with each allele of rs4519508 (C > T) (Table 1) that C allele of rs4519509 exhibits a global higher combination score with TFs. Bioinformatic prediction revealed that rs4519508 C > T reduced the binding affinity of STAT1 and destroyed the binding sites of E2F6. ChIP-seq evidence for E2F6 binding is shown in Supplementary Figure S4.

### 3.3. Analysis of Candidate TF Binding Elements Altered by rs4519508 C > T

To investigate the effect of rs4519508(C > T) on the binding of candidate TFs, we overexpressed the four TFs (pcDNA3.1-E2F4, pcDNA3.1-E2F6, pcDNA3.1-ETS1, and pcDNA3.1-STAT1), then conducted the luciferase assay.

**Figure 2:**
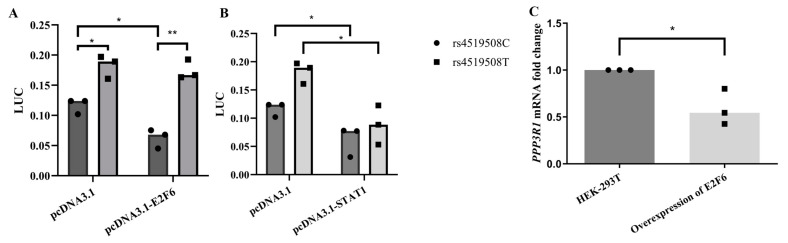
Rs4519508 (C > T) affected the binding of transcription factors. (**A**) Effects of rs4519508 on the transcriptional activity of *PPP3R1* with(out) the overexpression of E2F6. Compared with control group of pcDNA3.1, the overexpression of E2F6 significantly reduced the transcriptional activity level of constructs containing rs4519508C (*p* = 0.0126), while the reduction effect on rs4519508T was not statistically significant (*p* > 0.05). pcDNA3.1, basal level of luciferase activity transfected by rs4519608C or rs4519608T in pGL3-basic; pcDNA3.1-E2F6, luciferase activity with the overexpression of E2F6 transfected by pGL3-basic containing rs4519608C or rs4519608T. (**B**) Effects of rs4519508 on the transcriptional activity of *PPP3R1* with(out) the overexpression of STAT1. Both genotypes of rs4519508 had lower luciferase responses compared to control group without the overexpression of STAT1, and the drop was greater in the minor T allele of rs4519508. pcDNA3.1-STAT1, luciferase activity with the overexpression of STAT1 transfected by rs4519608C or rs4519608T in pGL3-basic. (**C**). qPCR results showed that the expression of PPP3R1 was significantly decreased by the overexpression of E2F6 (0.5-fold, *p* < 0.05). (*n* = 3) **, *p* < 0.01; *, *p* < 0.05. *p*-values were calculated using Student’s *t*-test.

The overexpression of E2F6 significantly reduced the luciferase gene expression for constructs containing rs4519508 C compared with the basal level, while the influence was not statistically significant in the presence of rs4519508 T (Figure 2A, Supplementary Table S5). QRT-PCR also showed that *PPP3R1* expression level was significantly decreased by the overexpression of E2F6 (0.5-fold; *p* < 0.05) (Figure 2C). For STAT1, both rs4519508 genotypes had lower luciferase responses than the control group, and the decrease was greater in the minor T allele than in the major C allele (Figure 2B). The results indicated that the binding of STAT1 suppressed transcriptional activity. The binding capacity of STAT1 was slightly impaired by the mutation from C to T at the rs4519508 site; thus, the polymorphism attenuated the inhibitory effect of STAT1. Experiments for E2F4 and ETS1 showed no significant results.

In summary, compared with other TFs, the mutation of rs4519508 C to T exerts a more significant alteration on the binding of E2F6.

### 3.4. E2F6 Binds to the PPP3R1 rs4519508 Site

**Figure 3:**
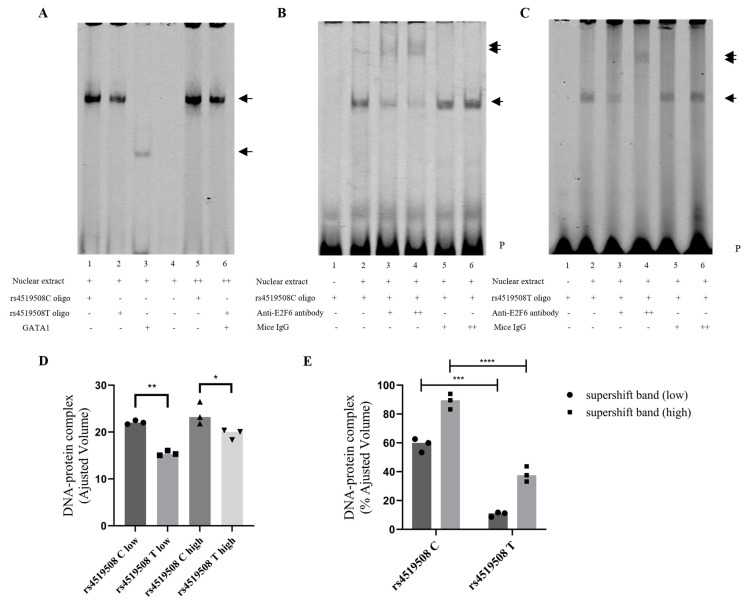
E2F6 binds to *PPP3R1* rs4519508 site in vitro. (**A**). EMSA was performed using heat-shocked nuclear extract from Jurkat T cells; arrows, DNA–protein complexes; P, free biotin-labeled probe. Lane 1 and 2 represent rs4519508C (wild-type) and rs4519508T (mutant) oligonucleotides, respectively, with 3 mg (low) of total nuclear extract. Lanes 3 and 4 represent positive and negative control serum with and without GATA1, respectively. Lanes 5 and 6 represent rs4519508C (wild-type) and rs4519508T (mutant) oligonucleotides, respectively, with 6 mg (high) of total nuclear extract. (**B**). Supershift assays with anti-E2F6 antiserum revealed the binding of E2F6 to the rs4519508C (wild-type). Arrows, DNA–protein complexes; double arrows, supershifted bands; P, free biotin-labeled probe. Lanes 1 and 2 show binding of DNA–protein complexes at rs4519508C; lanes 3 and 4 validate that E2F6 antibody eliminated the shift; lanes 5 and 6 used mice IgG as positive control. (**C**). Supershift assays with anti-E2F6 antiserum revealed the binding of E2F6 to the rs4519508T (mutant). Arrows, DNA–protein complexes; double arrows, supershifted bands; P, free biotin-labeled probe. Lanes 1 and 2 show the binding of DNA–protein complexes at rs4519508T; lanes 3 and 4 validate that E2F6 antibody eliminated the shift; lanes 5 and 6 used mice IgG as positive control. (**D**) Densitometry results for EMSA (**A**) are represented as adjusted volume for rs4519508C(T). (**E**) Densitometry results for supershifted bands (DNA-E2F6-antibody complex) (**B**,**C**) are represented as percent adjusted volume for rs4519508C(T) (compared with lane 2, respectively). (*n* = 3). ****, *p* < 0.0001, ***, *p* < 0.001, **, *p* < 0.01, *, *p* < 0.05. *p*-values were calculated using Student’s *t*-test.

We further tested whether E2F6 can bind to the *PPP3R1* rs4519508 site via EMSA. As shown in Figure 3A,D, there was a significant binding difference between the total nuclear extract of rs4519508 C and rs4519508 T oligonucleotides. The significant decrease in the protein–DNA complex indicates that the allelic variation influences the efficient binding between proteins. Moreover, supershift assays using anti-E2F6 antiserum demonstrated the binding of E2F6 to the wild-type of rs4519508, which suggests that E2F6 autoregulates transcription in *PPP3R1* promoter region (Figure 3B,E). We observed a specific protein complex bound to the rs4519508 C oligos, marked by a shift (double arrowhead); with addition of rs4519508 T oligos (Figure 3C,E) the combination was significantly weaker than that with rs4519508 C oligos, suggesting that the binding complex is specific for the wild-type allele ‘C’ of rs4519508.

Together, these results demonstrate that E2F6 can bind to the *PPP3R1* promoter region in vitro, and that this binding can be abolished by the mutation from C to T at the rs4519508 site (Supplementary Figure S5).

### 3.5. E2F6 Knockdown Reduced the Immunosuppressive Effect of TAC

The protein–protein interaction network for *PPP3R1* with a high STRING score threshold showed direct interactions between *PPP3R1* and key coding genes in the FKBP-CaN-NFAT pathway [41], the NFAT family, and immunophilin of CNI (Supplementary Figure S6). E2F6 binding to the *PPP3R1* promoter region may play a role in regulating the FKBP-CaN-NFAT pathway, and thus TAC efficacy.

**Figure 4:**
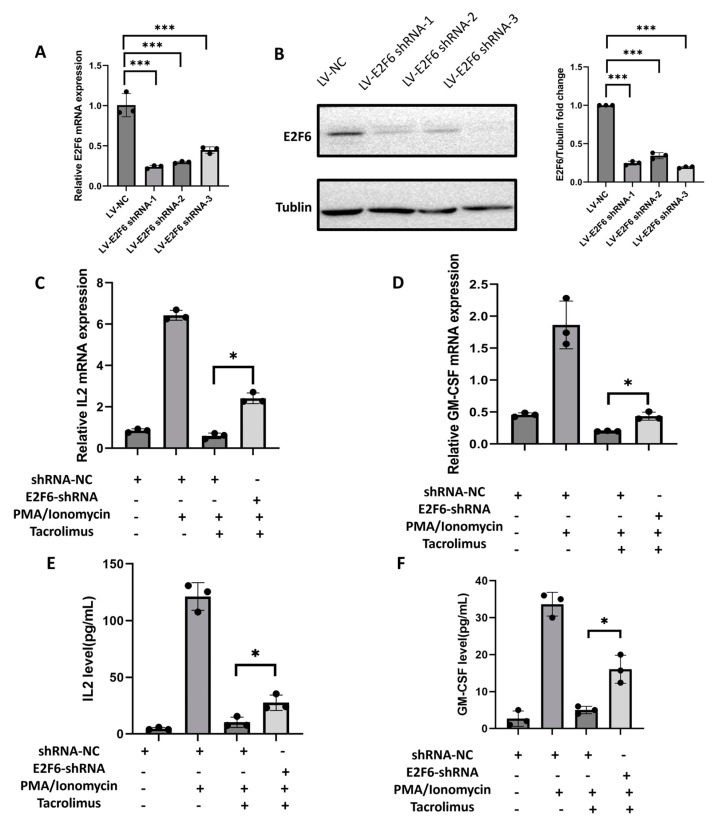
The effect of E2F6 knockdown on the immunosuppressive effect of tacrolimus. Real-time PCR analysis showed E2F6 mRNA expression (**A**) and Western blots verified E2F6 knockdown (**B**). Real-time PCR analysis of IL-2 mRNA expression (**C**,**D**) and GM-CSF mRNA expression (**E**,**F**) with ELISA analysis of IL-2 and GM-CSF. (*n* = 3). ***, *p* < 0.001; *, *p* < 0.05. *p*-values were calculated using Student’s *t*-test.

We further validated the effect of E2F6 on the immunosuppressive effect of TAC in E2F6 knockdown in Jurkat T cells. The lentivirus package was applied to construct E2F6-knowdown recombinant LVs (LV-E2F6-shRNA) and transduced with Jurkat T cells. As shown in Figure 4A,B, E2F6 mRNA and protein levels were downregulated by the transduction of LV-E2F6-shRNA. The full Western blots are presented in Supplementary Figure S7. In the presence of TAC, E2F6 knockout led to significantly higher IL-2 and GM-CSF mRNA levels compared with shRNA for control (*p* < 0.05) (Figure 4C,D). Meanwhile, the levels of IL-2 and GM-CSF production were notably higher in E2F6 knockout group than in control group (*p* < 0.05) (Figure 4E,F). The upregulation of IL-2 and downstream immune cytokine GM-CSF induced by the knockdown of E2F6 indicated that the immunosuppressive effect of TAC was weakened, which further validated that E2F6 acted as a repressor of PPP3R1, then inhibited the downstream immune activation of TAC.

## 4. Discussion

Genetic variations in PK and PD genes may account for inter-patient variability in clinical drug effects. Pharmacogenetic studies on TAC have mainly focused on the key genes involved in the metabolic process. However, few studies have investigated the genetic factors in the PD pathway of TAC. Here, we first identify that rs4519508 C > T in *PPP3R1* upregulate the expression by impairing the E2F6 binding affinity, thus attenuating TAC efficacy.

*PPP3R1*, coding gene of protein phosphatase 2 B regulatory subunit 1, also known as calcineurin B, participates in several important pathways, including the CaN-NFAT pathway, immune response, apoptosis, Ca^2+^ pathway, and MAPK signaling pathway. The protein–protein interaction network for *PPP3R1*, using a high STRING score threshold, showed direct interactions between *PPP3R1* and key coding genes in the FKBP-CaN-NFAT pathway [41], the NFAT family, and immunophilin of CNI. There is considerable evidence supporting the role of the CaN-NFAT signaling cascade (Gene Ontology: 0033173) (FDR = 1.99 × 10^−14^) in the immunosuppressive action of TAC [41,42]. Based on our previous association studies, we determined that *PPP3R1* rs875 (*p* = 0.037), rs4347819 (*p* = 0.045), and rs7560138 (*p* = 0.024) are significantly associated with allograft function or the incidence of TAC-induced acute rejection [3,19]. In addition, we found that *PPP3R1* rs875 T > C may affect TAC efficacy in IMN patients by mediating miR-582-5p binding to *PPP3R1* [15]. Previous studies have shown that SNPs in strong LD with the critical SNP could be functional in the mechanism of complex diseases [21,22,23,24,25], indicating the importance of studies on LD SNPs for the identification of causal mutations. Thus, aside from examining the critical SNP, one optional strategy is to analyze the functional role of SNPs that are in high LD with the lead SNPs [21,26].

*PPP3R1* promoter polymorphism, rs4519508 C > T, was identified through LD analysis to be in strong LD with the validated functional variant, rs875 (*r*^2^ > 0.9), and predicted to affect the binding affinity of E2F6 by using in silico analysis. We also compared the effects of the two LD SNPs and found that rs4519508 and rs875 had equivalent effects of increasing the luciferase activity. We found no significant synergistic effect between these two LD SNPs, which may be explained by a certain saturation effect at the presence of simultaneous mutations, while simultaneous mutation of these two polymorphisms may induce profound influence from the perspective of conservation. As the minor allele frequency of rs4519508 was 0.413 in Han Chinese in Beijing (China) and 0.50 in East Asian population, the function of this polymorphism is worthy of further investigation.

Molecular mechanism assays revealed that rs4519508 allelic variation impaired the binding affinity of E2F6, which repressed the *PPP3R1* expression, and thus influenced the immunosuppressive effect of TAC. We have figured out that the allelic variation of rs4519508 C > T significantly elevates PPP3R1 promoter activity (Figure 1A). Bioinformatic analysis indicates that the TF motif of E2F6 binds to the specific position of rs4519508 site and the binding could be destroyed by the mutation of rs4519508 C to T (Table 1). EMSA assays validated the binding of E2F6 to the rs4519508 C site and the binding was significantly weaker to the rs4519508 T site (Figure 3). Then, experiments verified that the overexpression of E2F6 significantly reduced the transcriptional activity and expression of PPP3R1 when the rs4519508 site presented as major C allele, and the reduction effect was not observed with the rs4519508 minor T allele (Figure 2A,C). That is to say, E2F6 acts as a repressor of PPP3R1; meanwhile, rs4519508 C > T impairs the binding of E2F6 to PPP3R1 promoter region, and thus the suppressive effect of E2F6 to PPP3R1 could be weakened with the mutation of rs4519508 site from C to T, so that PPP3R1 promoter activity is upregulated with rs4519508 C > T. Furthermore, our previous published study [15] have explored the effect of PPP3R1 knockdown on the immunosuppressive effect of TAC. In the presence of TAC, PPP3R1 siRNA led to significantly lower IL-2 and GM-CSF mRNA levels compared with scramble siRNA (*p* < 0.01). Consistently, the levels of IL-2 and GM-CSF production were notably lower in PPP3R1-siRNA group than in scramble-siRNA group (*p* < 0.001). The experimental data demonstrated that PPP3R1 knockdown enhanced the immunosuppressive effect of TAC. Since TAC exerts its immunosuppressive effect through targeting and inhibiting CaN, the knockdown of PPP3R1 downregulates the level of CaN, which generates synergistic effect with TAC. In this study, we further investigated the effect of E2F6 knockdown. The results showed that, in the presence of TAC, E2F6 knockout led to significantly higher IL-2 and GM-CSF mRNA levels compared with shRNA for control (*p* < 0.05) (Figure 4C,D). Also, the levels of IL-2 and GM-CSF production were notably higher in E2F6 knockout group than in control group (*p* < 0.05) (Figure 4E,F). The upregulation of IL-2 and downstream immune cytokine GM-CSF induced by the knockdown of E2F6 indicated that the immunosuppressive effect of TAC was weakened. Since the downregulation of PPP3R1 can further reduce the level of downstream immune cytokines inhibited by TAC, and the downregulation of E2F6 raises the level of downstream immune cytokines inhibited by TAC, we propose that E2F6 suppresses the expression of PPP3R1, while rs4519508 C > T impairs the binding of E2F6, and thus elevates the level of PPP3R1, so that the inhibition of the downstream immune cytokines by TAC is attenuated (Supplementary Figure S8).

E2F6, E2F transcription factor 6, is a member of the E2F family, which encodes a family of TFs which participate in the control of the cell cycle and interacts with a complex with chromatin-modifying factors [43,44,45,46]. Since the E2F6 protein lacks transactivation and tumor suppressor protein association domains, which are found in other family members, and contains a modular suppression domain functioning in the inhibition of transcription [43,47], E2F6 turned out to be a dominant-negative transcriptional repressor against other members of the E2F family [43].

Here we found that E2F6 participated in the downregulation of *PPP3R1*. Studies have demonstrated the repressive regulation of E2F6 in several pathways. E2F6 has been reported to repress growth-associated apoptosis of human hematopoietic progenitor cells [46], and to inhibit islet beta cell proliferation and participate in the pathological process of type I and II diabetes [48]. Meanwhile, E2F6 suppresses Dnmt3b recruitment to mediate germ-line gene silencing in murine somatic tissues [49], and downregulates MIR22HG [50] and lncRNA CASC2 [51] participating in laryngocarcinoma and gastric cancer progression, respectively.

Our study supports the regulatory association between the E2F6 and CaN signaling pathway, which participates in the immunosuppressive effect of TAC. With these observed phenotype changes, further investigation is required to determine the mechanism of this regulatory pathway. There are, however, several limitations in our study. Further clinical validation with a large cohort is required to verify the effect size of allelic variation in patients, and it is also essential to explore racial discrepancies across different populations. Clinical characteristics and other genetic and non-genetic confounding factors should be incorporated to evaluate the impact of allelic variation on TAC efficacy. Further investigation is essential to evaluate other potential transcriptional or epigenetic regulators that could influence TAC efficacy. Meanwhile, a more comprehensive approach that accounts for multiple genetic markers is required to determine a clearer understanding of individual variability in response to tacrolimus. Longer-term clinical observation studies are required to further evaluate the effect of rs4519508 in prolonged treatment of TAC. It is also greatly helpful to supplement our findings using patient-driven cells or tissues. Subsequent studies are expected to further consolidate the molecular mechanism.

## 5. Conclusions

This study is the first to report a *PPP3R1* promoter SNP rs4519508 (C > T) as a potential pharmacogenetic biomarker for TAC efficacy. Functional assays revealed that the mutation of rs4519508 from C to T impaired the TF binding site of E2F6, which worked as a repressor of *PPP3R1*, thus upregulating the expression of *PPP3R1* and subsequently affecting the downstream immune response of TAC. Our findings reported the potential regulatory role of a novel noncoding polymorphism, *PPP3R1* rs4519508, which may influence the TAC efficacy by affecting E2F6 binding and *PPP3R1* expression. Further study is required to figure out the molecular mechanism of these regulations.

## Figures and Tables

**Table 1:** Transcription factors prediction of *PPP3R1* rs4519508 by JASPAR and Cistrome.

Allele.	Start	End	Combination Score	Strand	TF	Brief Comment
C	5	15	0.8236	+	E2F4	E2F transcription factor 4, target of the transforming proteins of small DNA tumor viruses, important in the control of cell cycle and action of tumor suppressor proteins, altered by the mutation of C to T. The average binding score of the two predicted binding sites within rs4519508 C was equivalent to that within rs4519508 T, which suggested that the rs4519508 mutation has no major impact on the binding of E2F4.
C	6	16	0.9264	+	E2F4	
T	6	16	0.8724	+	E2F4	
C	6	16	0.8769	+	E2F6	E2F transcription factor 6, abolished when in allele T.
C	8	17	0.8256	+	ERG	ETS transcription factor ERG, abolished when in allele T, while ERG shows no expression in both HEK 293 T cells and blood cells, which reduce its value to validate.
C	8	17	0.8191	+	ETS1	ETS1: ETS proto-oncogene 1, transcription factor, two motifs in allele C and abolished when in allele T. ETS1 was predicted to bind to the genotype of the C allele alone, but ETS1 shows no expression in both HEK 293 T cells and blood cells, which reduce its value to validate.
C	1	10	0.8022	+	ETS1	
C	5	15	0.8559	+	STAT1	STAT1: signal transducer and activator of transcription 1, important for cell viability, altered by the mutation of C to T.
T	5	15	0.8006	+	STAT1	

## Data Availability

Data supporting the findings of this study are available within the article. Further data can be obtained upon reasonable request from the corresponding authors.

## Supplementary Materials

The following supporting information can be downloaded at: https://www.mdpi.com/article/10.3390/biomedicines12122896/s1, Table S1: Primer sequences in plasmid construction; Table S2: Primer sequences in TF-overexpression plasmid construction; Table S3: Primer sequences for qRT-PCR; Table S4: Comparative analysis between rs4519508C/T-rs875T/C combined recombinants; Table S5: Comparative analysis of TFs between different genotypes of rs4519508; Figure S1: Construction of pGL3-rs4519508C (wild-type), pGL3-rs4519508T (mutant), pGL3-rs875T (wild-type), and pGL3-rs875C (mutant); Figure S2: Construction of pGL3-rs4519508C-rs875T, pGL3-rs4519508T-rs875T, pGL3-rs4519508C-rs875C, and pGL3-rs4519508T-rs875C; Figure S3: Rs875 T > C significantly increases transcriptional activity of *PPP3R1*; Figure S4: Illustration of ChIP-seq evidence for E2F6 binding; Figure S5: A transcription factor binding site for E2F6 is predicted to be produced at 5′UTR of *PPP3R1*; Figure S6: Protein–protein interaction network for PPP3R1 depicting important protein interactions with other components in FKBP-CaN-NFAT pathway; Figure S7: Original Western blots for E2F6 knockdown and control; Figure S8: We propose that E2F6 suppresses expression of PPP3R1, while rs4519508 C > T impairs binding of E2F6, and thus elevates level of PPP3R1, so that inhibition of the downstream immune cytokines by TAC is attenuated; Figure S9: Sequence alignment for pGL3-rs4519508C(T) recombinant plasmids sequencing; Figure S10: Sequence alignment for pGL3-rs875T(C) recombinant plasmids sequencing; Figure S11: Agarose gel electrophoresis for pGL3-rs4519508C(T)-rs875T(C) (6157 bp) and pGL3-rs4519508C(T) (5759 bp) [15].

## Appendix A

### Supplementary Details for Plasmid Construction

The promoter region fragment (956 bp) of *PPP3R1* containing the rs4519508 major C allele was cloned into the dual luciferase reporter gene vector pGL3-basic using T4 DNA ligase (New England BioLabs, Boston, MA). The 959bp fragment was amplified from human cDNA using a PCR primer pair. The fragment was cloned into the *MluI* and *BglII* sites to obtain the recombinant vector pGL3-rs4519508C (5756 bp). The DH5α competent strain *E. coli* strain (TIANGEN Biotech, Beijing Co., Ltd. #CB101) was used during the transformation process. Then, the Q5^®^ Site-Directed Mutagenesis Kit (E0554; NEB, USA) was used to construct a site-directed mutant plasmid of rs4519508 T, named pGL3-rs4519508T. The mutagenic primers were designed using v1 (New England Biolabs, Beverly, MA, USA) an online NEB primer design supporting software. The two recombinants featured with rs4519508 C&T were verified by agarose gel electrophoresis and DNA sequencing as identical, except that they were different at the rs4519508 site (Supplementary Figure S9). In addition, we inserted the 3′UTR region fragment (401 bp) of *PPP3R1* containing rs875 T into the *XbaI* and *BamHI* sites of the dual luciferase reporter vector pGL3-promoter to build the recombinant vector pGL3-rs875T (5411 bp). The 401 bp fragment was amplified from human cDNA using a PCR primer pair. The Q5^®^ Site-Directed Mutagenesis kit (E0554; NEB, USA) was used to construct a site-directed mutant recombinant, named pGL3-rs875C, with the mutagenic primer designed by NEBaseChanger™ v1 (New England Biolabs, Beverly, MA, USA). The two recombinants featured with rs875 T&C were verified by DNA sequencing as identical, except that they were different at the rs875 site (Supplementary Figure S10). Supplementary Figure S1 illustrates the construction procedure of pGL3-rs4519508C (wild-type), pGL3-rs4519508T (mutant), pGL3-rs875T (wild-type), and pGL3-rs875C (mutant).

Next, the ClonExpress^®^ II One Step Cloning Kit (C112) (Vazyme Biotech Co., Ltd. Nanjing, China) was used to reassemble fragments containing rs4519508 C/T and rs875 T/C into one reporter vector, respectively. We inserted the 3′UTR fragment (401 bp) containing rs875T into pGL3-rs4519508C and pGL3-rs4519508T plasmids at the *XbaI* site with the primer pair designed by CE Design (http://www.vazyme.com, accessed on 13 September 2021), so two new recombinant plasmids were constructed, named pGL3-rs4519508C-rs875T and pGL3-rs4519508T-rs875T (6157 bp). Phanta^®^ Max Super-Fidelity DNA Polymerase (Vazyme, #P505, Nanjing, China) was used for PCR amplification, and the mutagenic primer pair of rs875 T > C was used to mutate the rs875 T site to rs875 C of these two new recombinants to obtain pGL3-rs4519508C-rs875C and pGL3-rs4519508T-rs875C plasmids (6157 bp). Four new reassembled constructs were verified as identical, except for the rs4519508 and rs875 sites, by agarose gel electrophoresis and DNA sequencing (Supplementary Figure S11). The primer pairs used for recombinant construction are presented in Supplementary Table S1. Supplementary Figure S2 presents the construction procedure of pGL3-rs4519508C-rs875T, pGL3-rs4519508T-rs875T, pGL3-rs4519508C-rs875C, and pGL3-rs4519508T-rs875C.

Moreover, we constructed overexpression (OE) vectors of four transcription factors: STAT1, E2F6, E2F4, and ETS1. The fragments of the coding sequence (CDS) of these four transcription factors were amplified from human cDNA and cloned into the pcDNA3.1 vector. The primer pairs used are presented in Supplementary Table S2. Four OE vectors were obtained and named pcDNA3.1-E2F4, pcDNA3.1-E2F6, pcDNA3.1-ETS1, and pcDNA3.1-STAT1. The nucleotide sequences of the recombinant vectors were verified using DNA sequencing.
