# Supplementary material for: PPP3R1 Promoter Polymorphism (Allelic Variation) Affects Tacrolimus Treatment Efficacy by Modulating E2F6 Binding Affinity
Source: Biomedicines. 2024 Dec 19;12(12):2896. doi: 10.3390/biomedicines12122896 (PMC11727355; doi:10.3390/biomedicines12122896)
Supplement: Supplementary file 1 [file biomedicines-12-02896-s001.zip › biomedicines-3309499-supplementary.pdf]

Supplementary Table S1 Primer sequences in Plasmid construction.

Product	Primer sequences	Tm (℃)	Accession Number	Product Length (bp)	Design
PPP3R1 promoter fragm--ent	Forward：5’-ATACGCGTGGATCCAAGTCGGTACACGG-3’<br>Reverse：5’-ATAGATCTCTCACCATTTTGCTCGGCG-3’	59.1	NC_000002.12	956	PrimerBlast
Sanger sequencing for rs4519508	Forward：5’-TTTTCGTGCCTTGAGGTTGC-3’<br>Reverse：5’-TGGCTCGGAGAAGTGTTGC-3’	60	NC_000002.12	377	PrimerBlast
Site-directed mutant plas--mid of rs4519508C>T	Forward：5’-CCCGAGTTTC**C**CGGCCGTCTTCG-3’<br>Reverse：5’-GGCTGCAGCCTCGCGCTC-3’	72	/	377	NEBaseChanger
PPP3R1 3'UTR fragment	Forward：5’-ggTCTAGACTCTTATCAGAGAGTACCACCCA-3’<br>Reverse：5’-gccGGATCCGTTTCTGAATCAAGTATATTAA-3’	56.3	NC_000002.12	401	reference(Zhu et al., 2018)
Sanger sequencing for rs875	Forward：5’-CTCTTATCAGAGAGTACCACCCA-3’<br>Reverse：5’-GTTTCTGAATCAAGTATATTAA-3’	56	NC_000002.12	265	reference(Zhu et al., 2018)
Site-directed mutant plas--mid of rs875T>C	Forward：5’-CCCGAGTTTC**C**CGGCCGTCTTCG-3’<br>Reverse：5’-GGCTGCAGCCTCGCGCTC-3’	56	/	265	NEBaseChanger
Insertion of *PPP3R1* 3'UTR fragment	Forward: 5’-agatcgccgtgtaattctagaCTCTTATCAGAGAGTACCACCCAACA-3’<br>Reverse: 5’-gccggccgccccgactctagaGTTTCTGAATCAAGTATATTAAATTAAAGCC-3’	61	/	6157	ClonExpress II

Supplementary Table S2 Primer sequences in TF-overexpression plasmid construction.

Product	Primer sequences	Enzyme	Length(bp)	Tm (℃)	Accession Number	Exon location	CDS location
E2F4 CDS fragment, PCR from pcDNA3-E2F4 plasmid	Forward: 5’-ct<u>TCTAGA</u>aggaacggaagcggaagtg-3’<br>Reverse: 5’-tt<u>GGATCC</u>tcagaggttgagaacaggcacatc-3’	*XbaI*<br>*BamHI*	1331	60	NM_001950.4	1..208	74..1315
E2F6 CDS fragment, PCR from human DNA	Forward: 5’-ac<u>TCTAGA</u>caaatctggcttgctgggctag-3’<br>Reverse: 5’-at<u>GGATCC</u>gccatcagttgcttacttcaagc-3’	*XbaI*<br>*BamHI*	816	60	NM_198256.4	1..390	283..1128
ETS1 CDS fragment, PCR from pDONR223_ETS1_WT plasmid	Forward: 5’-tct<u>GCTAGC</u>agttggaaagagaccacagac-3’<br>Reverse: 5’-ataataat<u>GCGGCCGC</u>tcactcgtcggcatctgg-3’	*NheI*<br>*NotI*	1531	57	NM_001143820.2	72..154	86..1543
ERG CDS fragment, PCR from human DNA	Forward: 5’-tct<u>GCTAGC</u>aagggcaactaaagccgtca-3’<br>Reverse: 5’-atctgtat<u>GCGGCCGC</u>ttagtagtaagtgcccagatgagaag-3’	*NheI*<br>*NotI*	1547	58	NM_182918.4	1..115	98..1537
STAT1 CDS fragment, PCR from human DNA	Forward: 5’-tat<u>GCTAGC</u>CTAACGTGCTGTGCGTAGCTG-3’<br>Reverse:<br>5’-ataataat<u>GCGGCCGC</u>GAAAACTGTCGCCAGAGAAGATG-3’	*NheI*<br>*NotI*	2193	60	NM_007315.4	309..437	310..2562
Insertion of E2F4 CDS fragment, Homologous recombination	Forward:<br>5’-gccctctagactcgagcggccgcAGGAACGGAAGCGGAAGTG-3’<br>Reverse:	/	/	60.2	NM_001950.4	1..208	74..1315

	5’-cttggtaccgagctcggatccTCAGAGGTTGAGAACAGGCACA-3’						
Insertion of E2F6 CDS fragment, Homologous recombination	Forward: 5’-gccctctagactcgagcggccgcCAAATCTGGCTTGCTGGGC-3’ Reverse: 5’-cttggtaccgagctcggatccGCCATCAGTTGCTTACTTCAAGC-3’	/	/	61.2	NM_198256.4	1..390	283..1128
Insertion of ETS1 CDS fragment, Homologous recombination	Forward: 5’-gggagacccaagctggctagcTCCTGGCACCATGAAGGCG-3’ Reverse: 5’-atatccagcacagtggcggccgcTCACTCGTCGGCATCTGGC-3’	/	/	63	NM_001143820.2	72..154	86..1543

Supplementary Table S3 Primer sequences for qRT-PCR.

Target mRNA	Primer sequences	Accession Number	Exon location	CDS location
PPP3R1	Forward： 5’-GTGCTCACACTTTGATGCGG-3’<br>Reverse： 5’-TCTCCATTCCCATCTGTGTCG-3’	NM_000945.4	1..408	406..918
E2F4	Forward： 5’-TCCGGACCCAACCCTTCTAC-3’<br>Reverse： 5’-GGGGTGGAGAAAGACGAAGC-3’	NM_001950.4	1..208	74..1315
E2F6	Forward： 5’-TCCATGAACAGATCGTCATTGC-3’<br>Reverse： 5’-TCCGTTGGTGCTCCTTATGTG-3’	NM_198256.4	1..390	283..1128
ERG	Forward： 5’-AGCTACAACGCCGACATCC-3’<br>Reverse： 5’-GACGCTGGTCTTCAGTTTTGG-3’	NM_182918.4	1..115	98..1537

ETS1	Forward：5’-ACTTTGTGGATTCTGCTGGGA-3’<br>Reverse：5’-CAAAAGGGGTAGCAAGGTCT-3’	NM_001143820.2	72..154	86..1543
STAT1	Forward：5’-CCATCCTTTGGTACAACATGC-3’<br>Reverse：5’-TGCACATGGTGGAGTCAGG-3’	NM_007315.4	309..437	310..2562
IL-2	Forward：5’-GACCCAGGGACTTAATCAGCA-3’<br>Reverse：5’-AATGGTTGCTGTCTCATCAGC-3’	NM_000586.4	1..432	286..747
GM-CSF	Forward：5’-GCATGTGAATGCCATCCAGG-3’<br>Reverse：5’-CCTGCTTGTACAGCTCCAGG-3’	NM_000758.4	1..194	36..470
GAPDH	Forward：5’-GAAGGTGAAGGTCGGAGTC-3’<br>Reverse：5’-GAAGATGGTGATGGGATTTC-3’	NM_002046.7	54..105	77..1084

Supplementary Table S4 Comparative analysis between rs4519508C/T-rs875T/C combined recombinants.

Group wise results		P value	Sig.	t	95% CI
Col1vs.Col2<br>wt-wt vs. mut-wt	rs4519508C-rs875T<br>vs.<br>rs4519508T-rs875T	3.727E-05	****	10	(0.5479, 0.8689)
Col1vs.Col3<br>wt-wt vs. wt-mut	rs4519508C-rs875T<br>vs.<br>rs4519508C-rs875C	0.0002	***	9.873	(0.4949, 0.8434)
Col1vs.Col4<br>wt-wt vs. mut-mut	rs4519508C-rs875T<br>vs.<br>rs4519508T-rs875C	0.0003	***	8.796	(0.5046, 0.9214)
Col2vs.Col3<br>mut-wt vs. wt-mut	rs4519508C-rs875T<br>vs.<br>rs4519508C-rs875C	0.6265	/	0.518	(-0.2343, 0.1557)
Col2vs.Col4<br>mut-wt vs. mut-mut	rs4519508C-rs875T<br>vs.<br>rs4519508T-rs875C	0.9607	/	0.052	(-0.2215, 0.2306)
Col3vs.Col4<br>wt-mut vs. mut-mut	rs4519508C-rs875C<br>vs.<br>rs4519508T-rs875C	0.668	/	0.4622	(-0.2196, 0.3073)

Col, column in Figure 4; wt, wild-type; mut, mutant type. Col1, rs4519508C-rs875T (wt-wt); Col2, rs4519508T-rs875T (mut-wt); Col3, rs4519508C-rs875C (wt-mut); Col4, rs4519508T-rs875C (mut-mut). ***, P < 0.001; **, $P < 0.01$; *, $P < 0.05$. P values were calculated by Student's t-test.

Supplementary Table S5 Comparative analysis of TFs between different genotypes of rs4519508.

Group wise results of Co-transfection		P value	Sig.	t	95% CI
Control	rs4519508C<br>rs4519508T	0.0141	*	4.167	(0.0206, 0.1029)
E2F6	rs4519508C<br>rs4519508T	0.001	**	8.576	(0.0753, 0.1474)
rs4519508C	Control<br>E2F6	0.0126	*	4.301	(-0.095, -0.0205)
rs4519508T	Control<br>E2F6	0.606	/	0.559	(0.04814, 0.032)
STAT1	rs4519508C<br>rs4519508T	0.3622	/	1.028	(-0.0443,0.0963)
rs4519508C	Control<br>STAT1	0.0332	*	3.189	(-0.111, -0.0076)
rs4519508T	Control<br>STAT1	0.0147	*	4.113	(-0.1578, -0.0306)

Wt, pGL3-rs4519508C; Mut, pGL3-rs4519508T; STAT1, pcDNA3.1-STAT1; E2F6, pcDNA3.1-E2F6; E2F4, pcDNA3.1-E2F4; ETS1, pcDNA3.1-ETS1. **, $P < 0.01$; *, $P < 0.05$. P values were calculated by Student's t-test.

Supplementary Figure S1 Construction of pGL3-rs4519508C (wild-type), pGL3-rs4519508T (mutant), pGL3-rs875T (wild-type), and pGL3-rs875C (mutant).

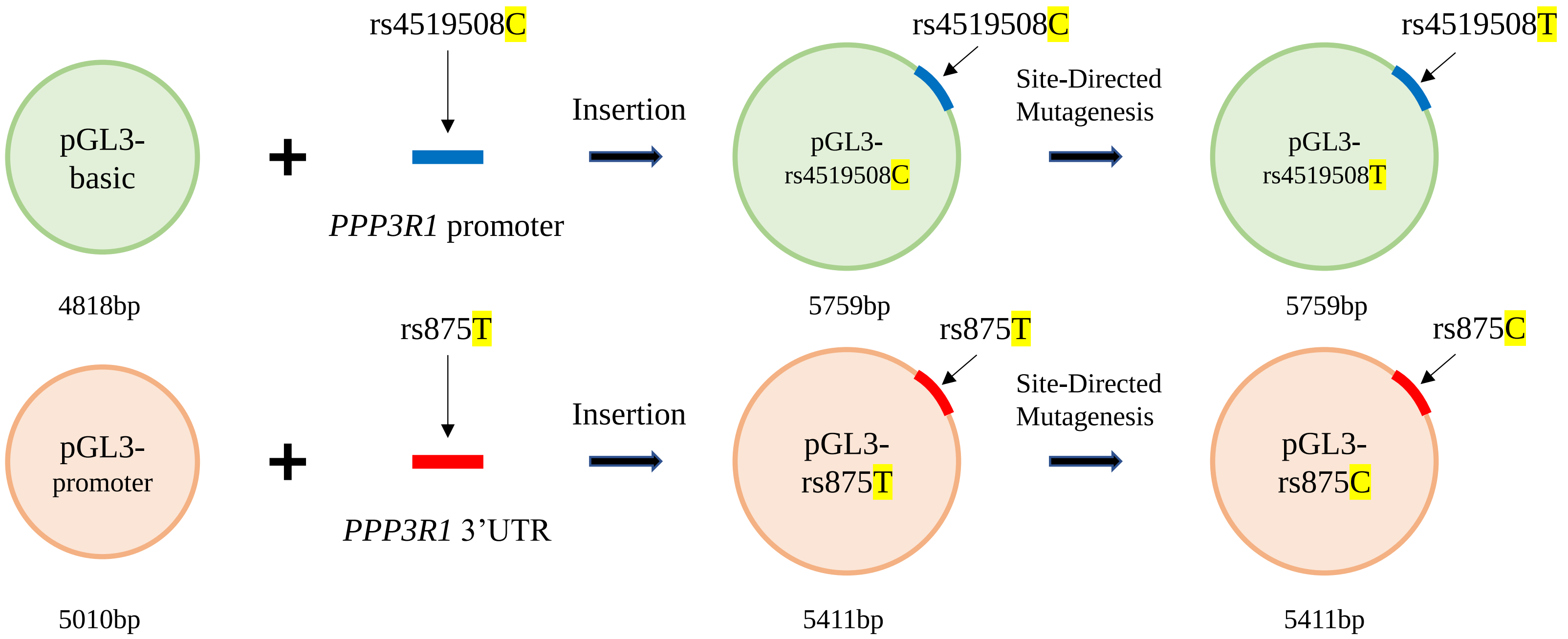

Supplementary Figure S2 Construction of pGL3-rs4519508C-rs875T, pGL3-rs4519508T-rs875T, pGL3-rs4519508C-rs875C, pGL3-rs4519508T-rs875C.

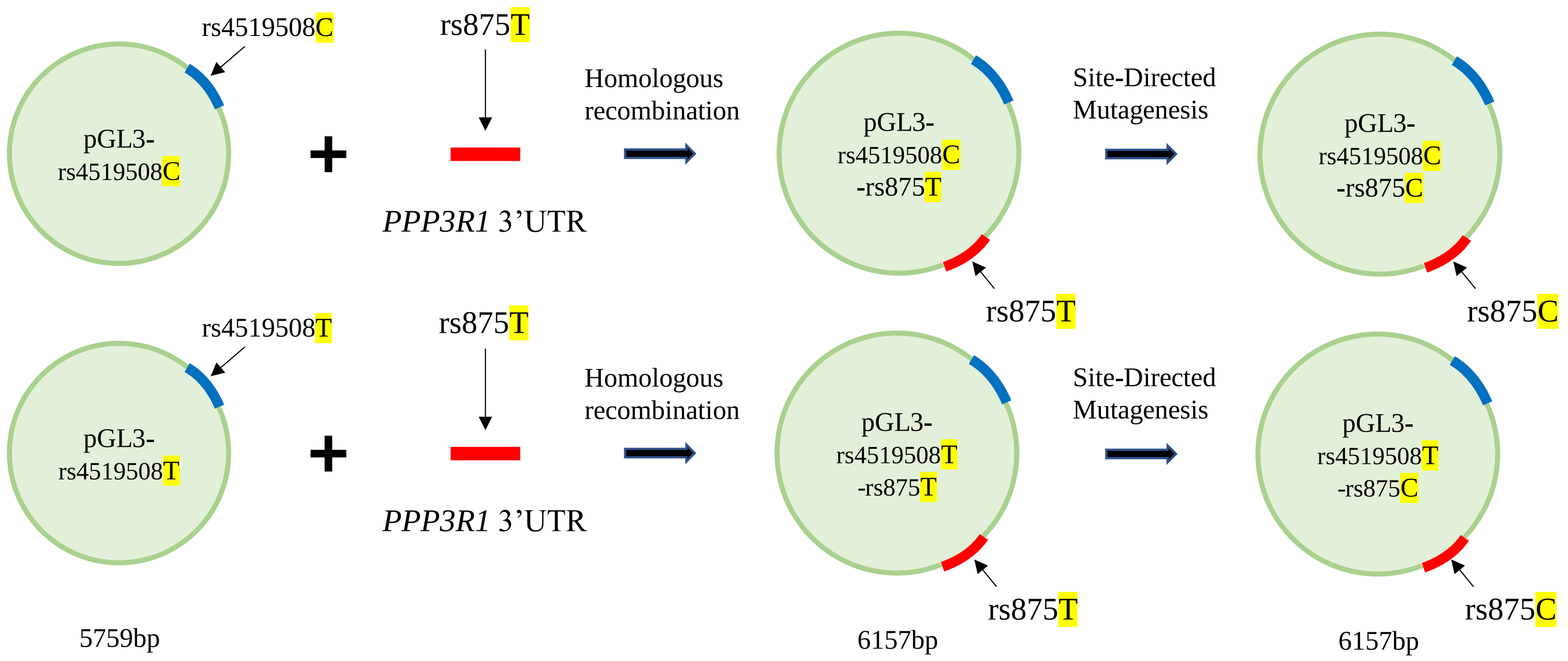

Supplementary Figure S3 Rs875 T>C significantly increases the transcriptional activity of *PPP3R1*.

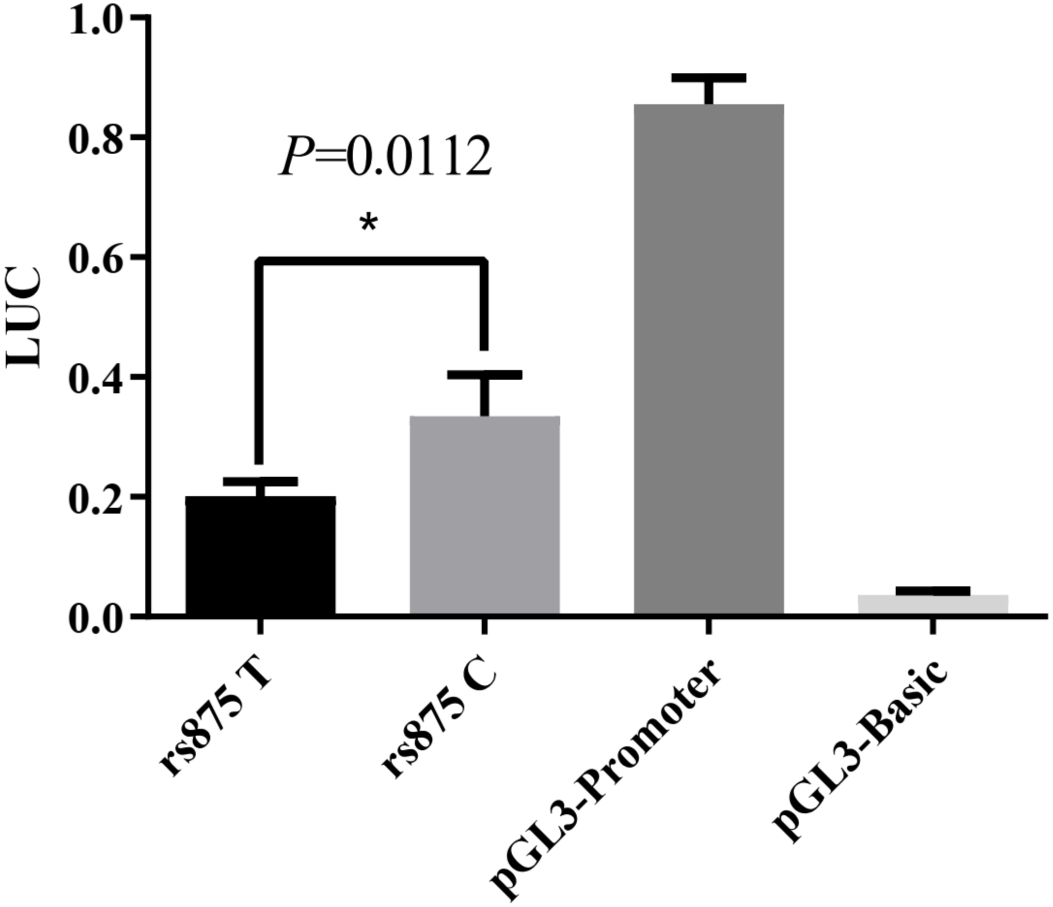

The pGL3-rs875C had significantly higher luciferase activity than the pGL3-rs875T (P = 0.0112). The pGL3-basic and pGL3-promoter reporter plasmids were as controls. Rs875 T, **pGL3-rs875T;** rs875 C, **pGL3-rs875C** promoter reporter recombinant vectors**.** *, P < 0.05. P values were calculated by Student's t-test.

Supplementary Figure S4 Illustration of Chip-seq evidence for E2F6 binding.

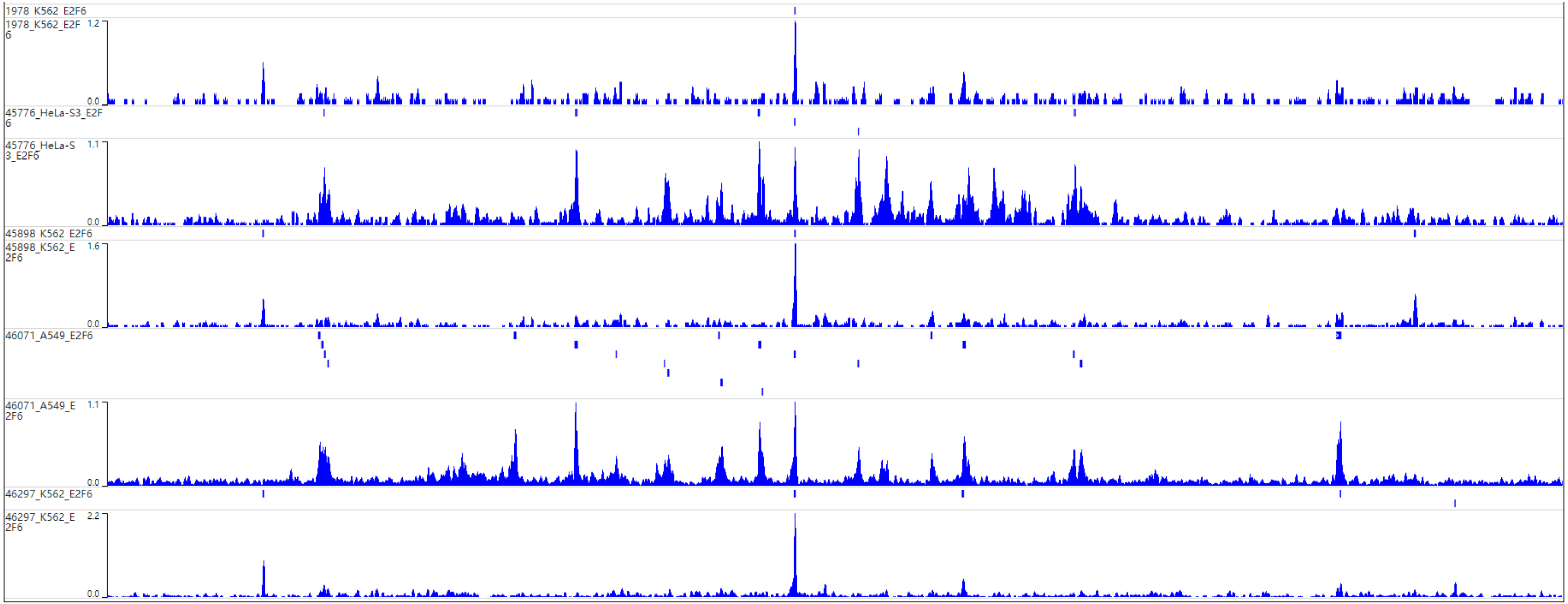

Copyright © 2018-2021 Washington University in St. Louis. All rights reserved.
Developed by the Wang Lab
Terms and Conditions of Use

Supplementary Figure S5 A transcription factor binding site for E2F6 is predicted to be produced at the 5′UTR of *PPP3R1*.

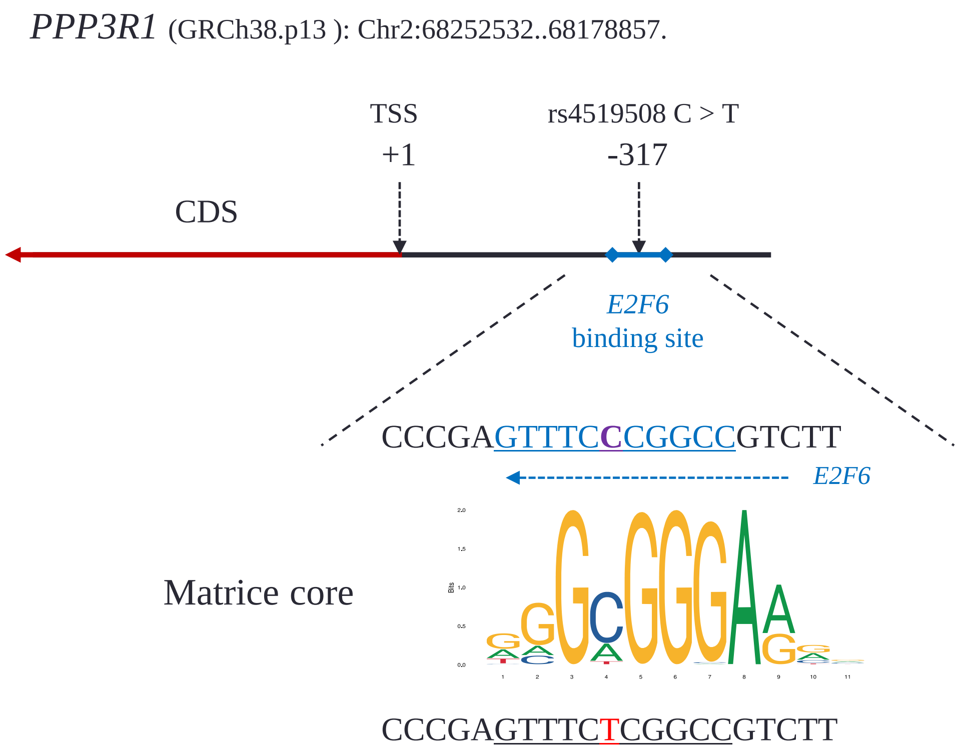

Supplementary Figure S6 Protein-protein interaction network for PPP3R1 depicting important protein interactions with other components in FKBP-CaN-NFAT pathway.

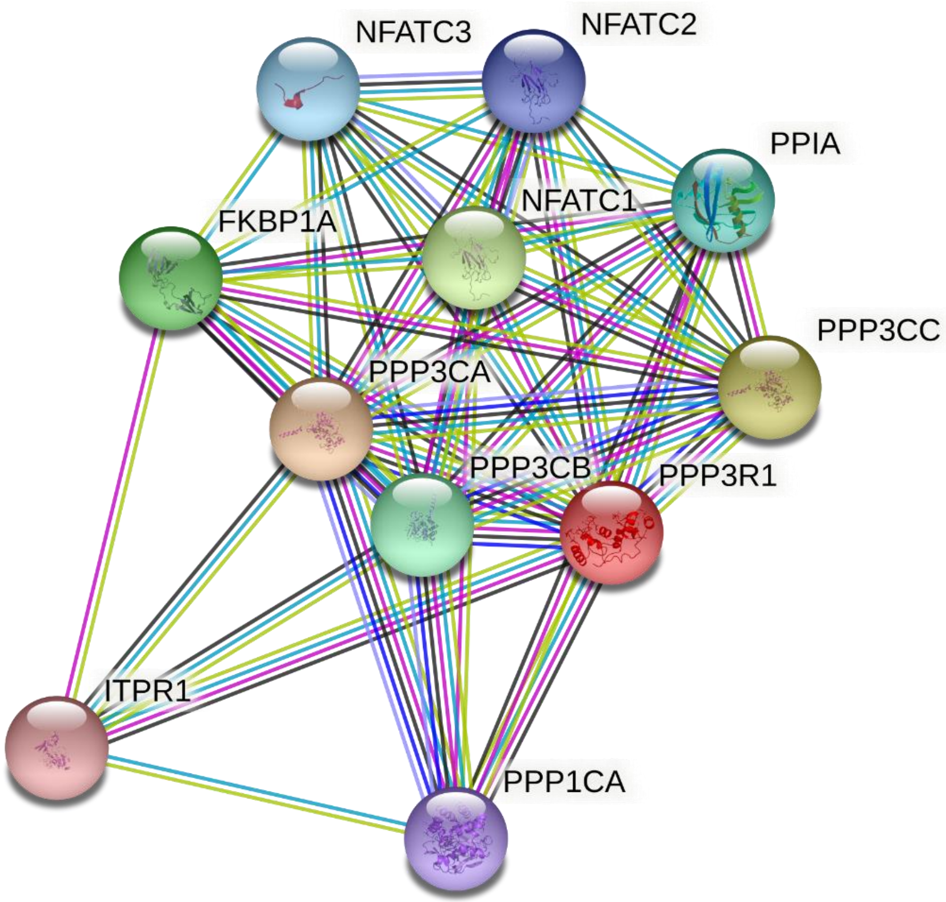

Supplementary Figure S7 The original western blots for E2F6 knockdown and control.

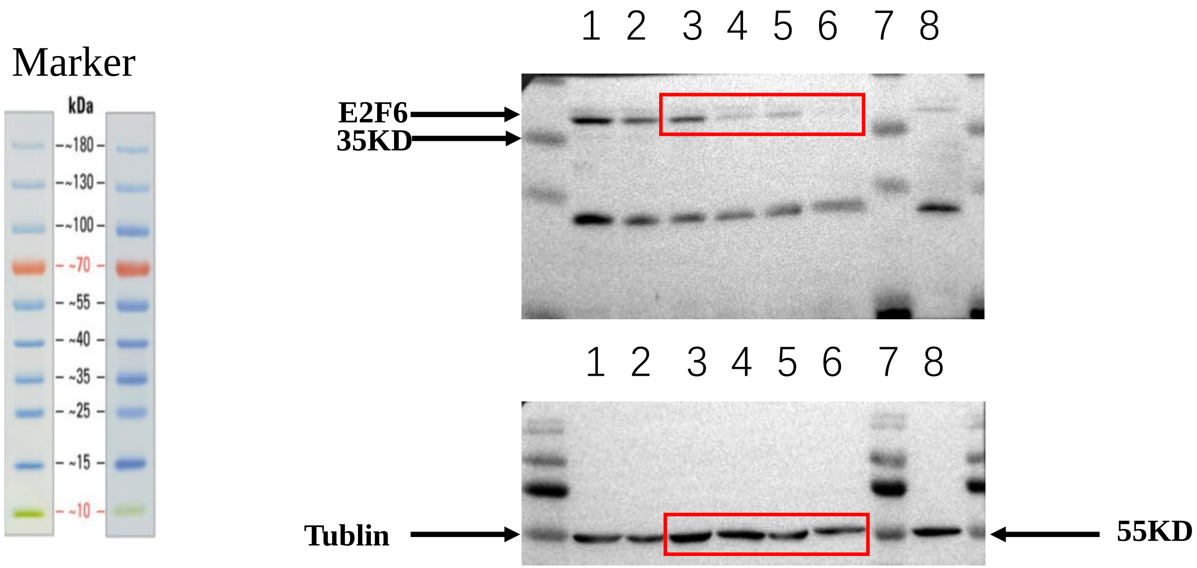

1:BLANK 2:LV-NC1 3:LV-NC2 4:LV-shRNA1 5:LV-shRNA-2 6:LV-shRNA-3 7:Marker 8:HepG2(WB positive control)

Supplementary Figure S8

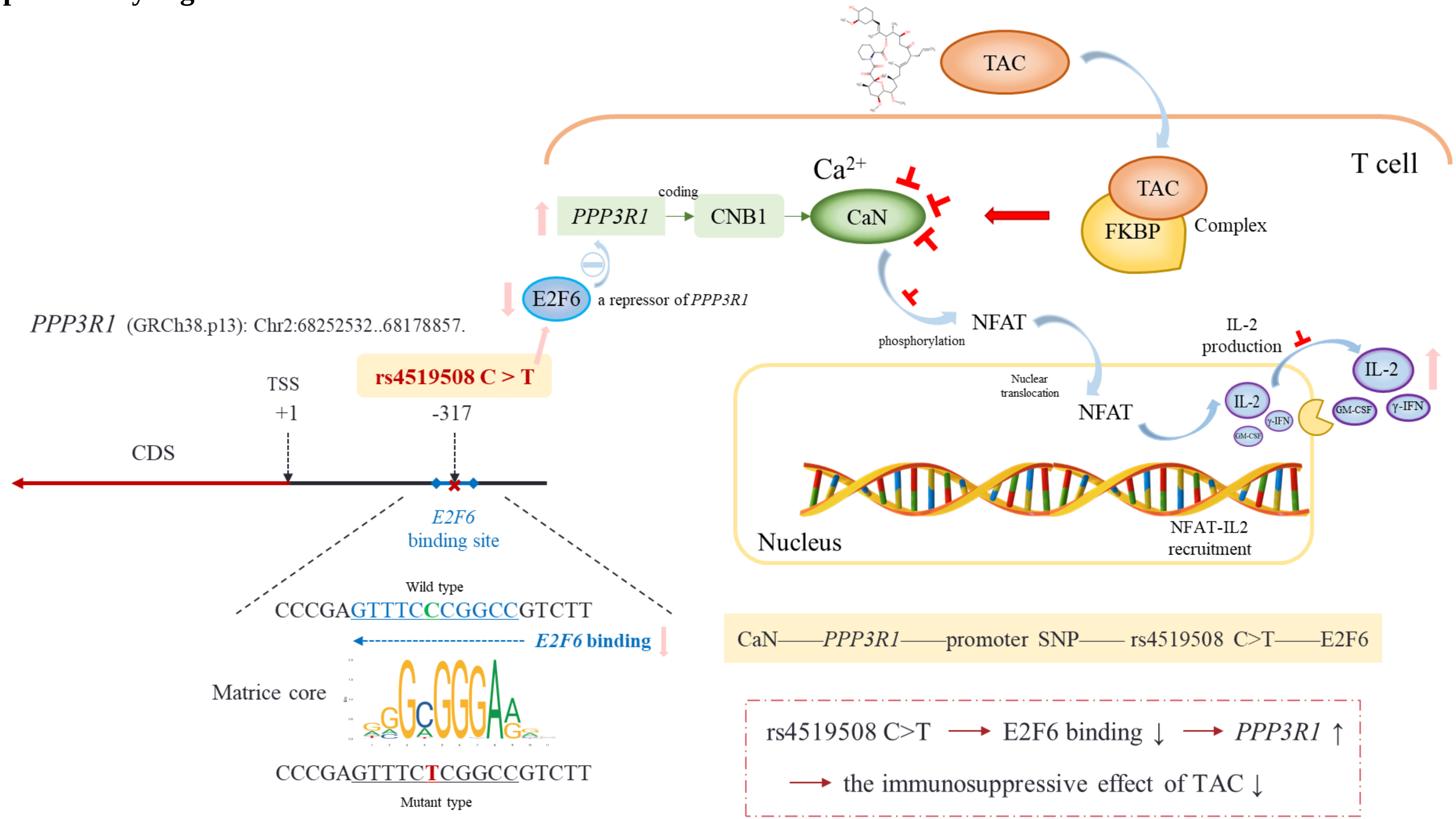

Supplementary Figure S9 Sequence alignment for pGL3-rs4519508C(T) recombinant plasmids sequencing.

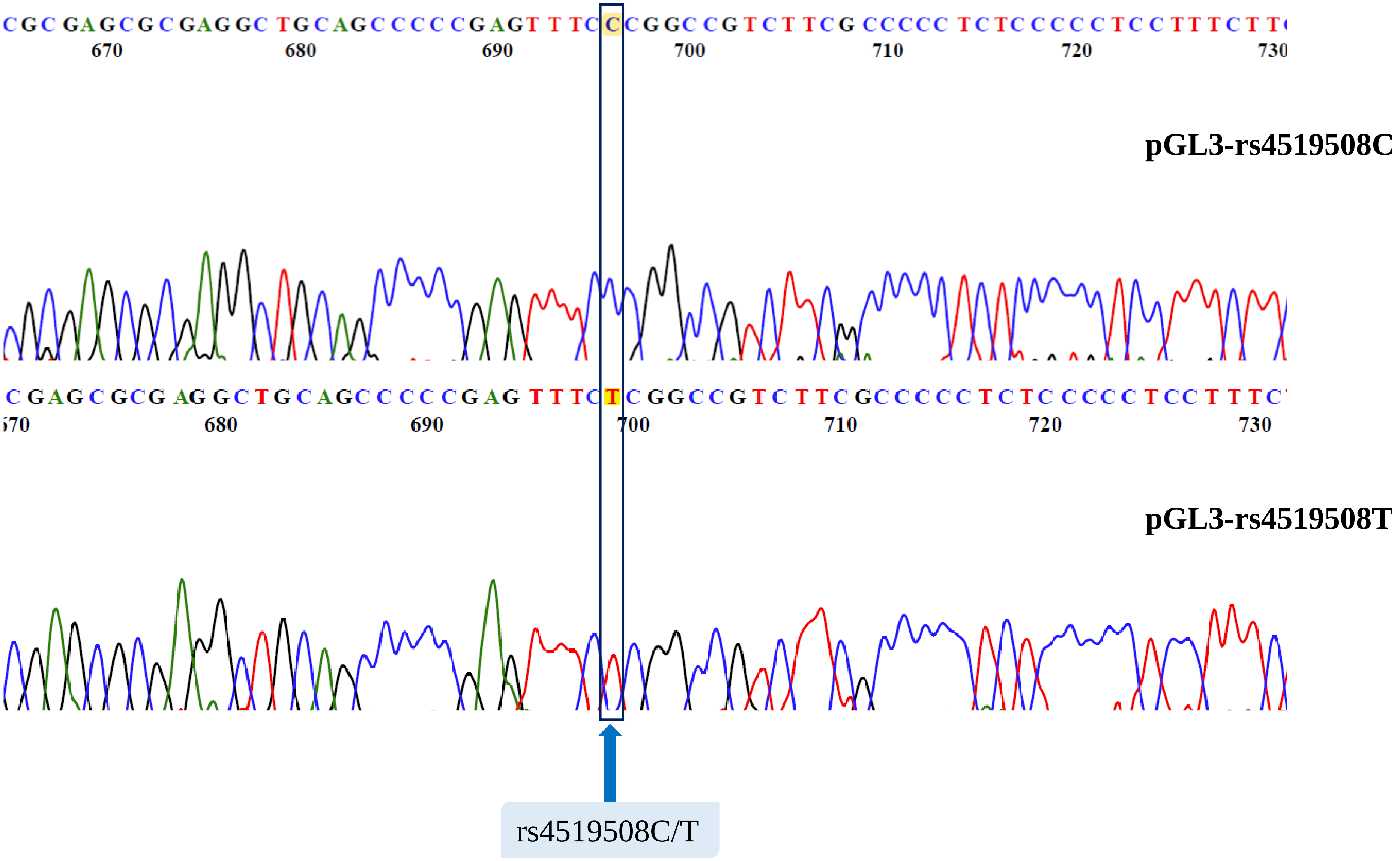

Supplementary Figure S10 Sequence alignment for pGL3-rs875T(C) recombinant plasmids sequencing.

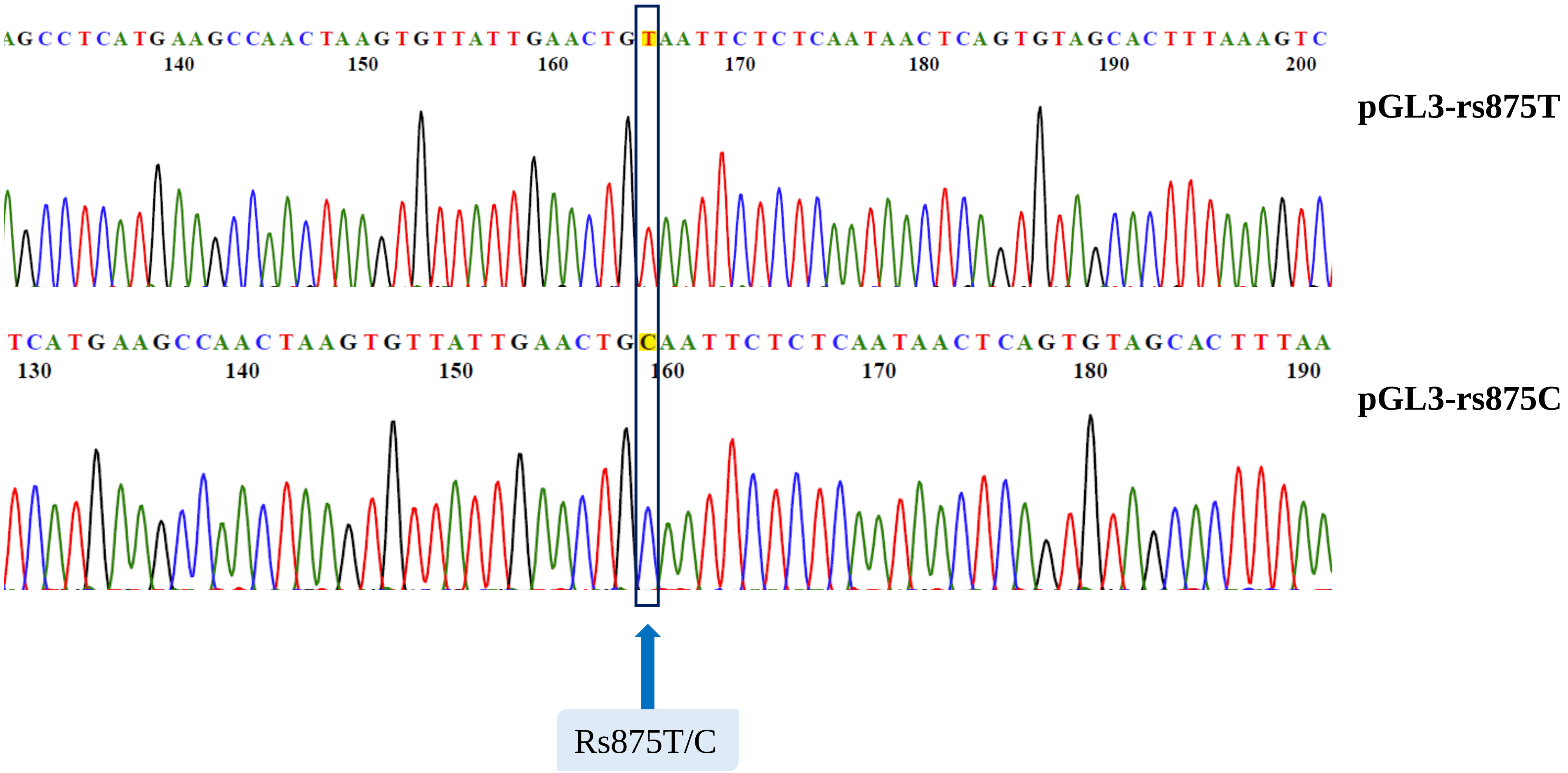

Supplementary Figure S11 Agarose gel electrophoresis for pGL3-rs4519508C(T)-rs875T(C) (6157bp) and pGL3-rs4519508C(T) (5759bp).

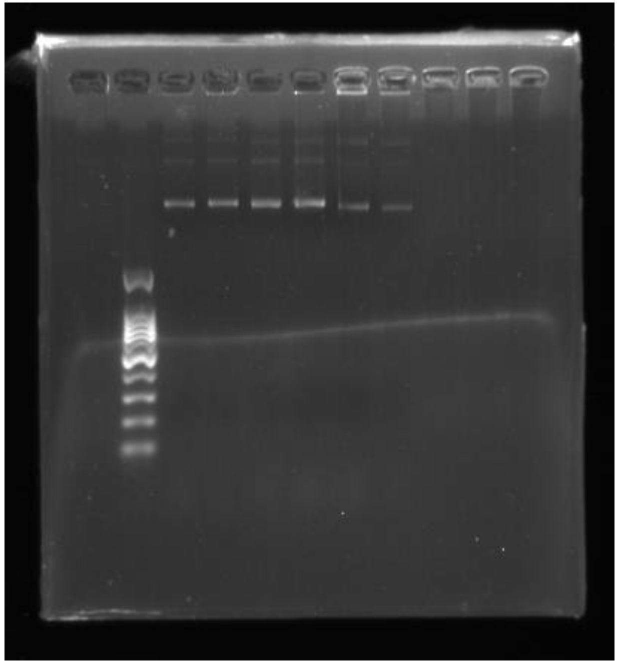

Lane1, 100bp ladder; Lane2, pGL3-rs4519508C-rs875T recombinant plasmid; Lane3, pGL3-rs4519508T-rs875T recombinant plasmid; Lane4, pGL3-rs4519508C-rs875C recombinant plasmid; Lane5, pGL3-rs4519508T-rs875C recombinant plasmid; Lane6, pGL3-rs4519508C recombinant plasmid; Lane7, pGL3-rs4519508T recombinant plasmid. The former four recombinant plasmids (pGL3-rs4519508C(T)-rs875T(C)) is 6157bp in length; The latter two recombinant plasmids (pGL3-rs4519508C(T)) is 5759bp in length.
